# Role of Chemically Functionalization of Bamboo Fibers on Polyethylene-Based Composite Performance: A Solution for Recycling

**DOI:** 10.3390/polym13152564

**Published:** 2021-07-31

**Authors:** Meisam Kouhi, Simona Butan, Yang Li, Elias Shakour, Mihaela Banu

**Affiliations:** 1 Department of Mechanical Engineering, University of Michigan, 2350 Hayward Street, Ann Arbor, MI 48109, USA; mason.kouhi@yanfeng.com (M.K.); mbanu@umich.edu (M.B.); 2 Yanfeng International, 41935 W 12 Mile Rd., Novi, MI 48377, USA; 3 Department of Chemistry, Physics and Environment, Dunarea de Jos University of Galati, 111 Domneasca Street, 800201 Galati, Romania; Simona.Patriche@ugal.ro; 4 School of Materials Science and Engineering, Tianjin University, 135 Yaguan Road, Tianjin 300354, China; 5 BASF Corporation, Composite Technologies, 1609 Biddle Avenue, Wyandotte, MI 48192, USA; elias.shakour@basf.com

**Keywords:** long bamboo fibers, low-density polyethylene, interface, mechanical properties

## Abstract

Low-density polyethylene is the most common polymer for manufacturing containers, bottles, tubes, plastic bags, computer components and so on. There is an urgent need to find solutions for its recycling and reintegration in high volume production components such as non-structural auto applications. The reinforcement of recycled low-density polyethylene with natural fibers represents a solution for the re-use of the recycled low-density polyethylene. However, there is a lack of understanding of how the natural fibers influence the behavior of the bare low-density polyethylene, and furthermore, how the interface between the fibers and the matrix can be controlled in composite to obtain the designed toughness, strength, stiffness and damping. In this sense, the study presents an in-depth analysis of the behavior of three coupling agents used in the chemically functionalized bamboo fibers interface for reinforcing low-density polyethylene composites. Through mechanical tests, the mechanical properties are determined and compared and finally, a correlation between the viscous behavior of the resulted composites and the toughening mechanism is proposed. The conclusion of the study enables a flexible design of polymer composite components fabricated of recycled and non-recycled low-density polyethylene and natural fibers.

## 1. Introduction

Low-density polyethylene (LDPE) is one of the most used thermoplastic polymers for food and non-food packaging purposes because of its low strength and increased foldability. LDPE packaging products lacks the end-of-life management jeopardizing the planet. For example, billions of LDPE water bottles annually end up in landfill. One rapid solution to overcome this challenge is to re-use them in high volume production components for transportation systems such as non-structural parts (i.e., Honda uses coconut composites as fillers of the interior doors, Mercedes S-Class has 27 components fabricated of such composites [1]). Additionally, an alternative to glass fiber or even carbon fiber composites are foreseen through the mass use of natural fibers in polymer composite components, thus leading to not only weight and cost reduction but also offering eco-friendly solutions. Despite of their relatively lower strength compared to synthetic fibers, the specific properties of the natural fiber composites are high due to the low density of the fibers (natural fibers density ~0.6–1.5 g/cm^3,^ compared to glass fibers density ~2.5 g/cm^3^) [2]. However, despite these potential advantages, big challenges were reported for natural fiber composites related to the unpredictable anisotropy of the properties, the capacity to undergo and sustain loadings and durability [2,3,4].

Among the developed and utilized natural fiber composites, it appears that the development and utilization of bamboo fiber composites has not received significant attention for the specified applications [5,6]. Bamboo culms are among the most promising precursors for extracting natural stand-alone fibers because of their exceptional properties and rapid growth rate. In addition to remarkable mechanical behavior, the moisture adsorption and desorption of bamboo fibers is the best of all known natural fibers. Bamboo fibers demonstrate a remarkable ultraviolet resistance (almost 20 times higher than that of cotton fibers) which is due to their sodium copper chlorophyllin content [7]. The research was mainly focused on the reinforcement of the thermoplastic polymers with short bamboo fibers (e.g., shredded bamboo culms) leading to mechanical properties required by non-structural components such as fillers. Limited research was oriented on the reinforcement of thermoplastic polymers with long bamboo fibers which have advantages over short fibers due to an enhanced capacity of load transfer leading to a high potential for manufacturing semi-structural parts [2,8,9,10,11]. High performance in load bearing composites relies on bonding at the interface between the fiber and the matrix. In the case of natural fibers, bonding with thermoplastic matrices is not yet controlled to allow the flexible design of components with different functionality. Addressing this niche in the context of the recyclability of LDPE and creating new environmentally friendly car components, the study focuses on the assessment of the impact of the interface on the mechanical properties of the LDPE long bamboo fibers (BFs) composites. Different chemically induced modifications of the fiber surfaces are generated for analyzing their impact on stiffness, strength and toughness.

The details of this experimental study are presented in the following sections: materials and methods, results and discussion and conclusion.

## 2. Materials and Methods

### 2.1. Bamboo Fibers Extraction

A newly proposed mechano-chemical processing was utilized to facilitate the extraction of the BFs and maintain their advantageous mechanical behavior (U.S. Patent No.10184215). Bamboo strips of 10 mm width × 300 mm length were cut from a dried moso bamboo culm and then soaked in a 2% sodium hydroxide solution (Fisher Scientific, Cleveland, OH, USA) for 24 h. The alkali-treated bamboo strips were subsequently soaked in a 1% hydrochloric acid solution (Fisher Scientific) for 3 h until they showed no reactivity to alkalinity. To further separate the hemicellulose and lignin, after washing with distilled water, the alkali-treated bamboo strips were placed in a steam autoclave, where they were saturated with overheated steam. The long BFs, were finally extracted from the treated strips through a fine combing process and subsequently dried in an air oven at 80 °C. Further, these fibers were used to test an interface formation in manufacturing low-density polyethylene reinforced composites.

The extracted long bamboo fibers (BFs) are bundles of 10–20 elementary fibers with a diameter of approximately 20–30 µm each, characterized by a length of 200–300 mm and leading to an aspect ratio (length to bundle diameter) of 1000.

**Figure 1:**
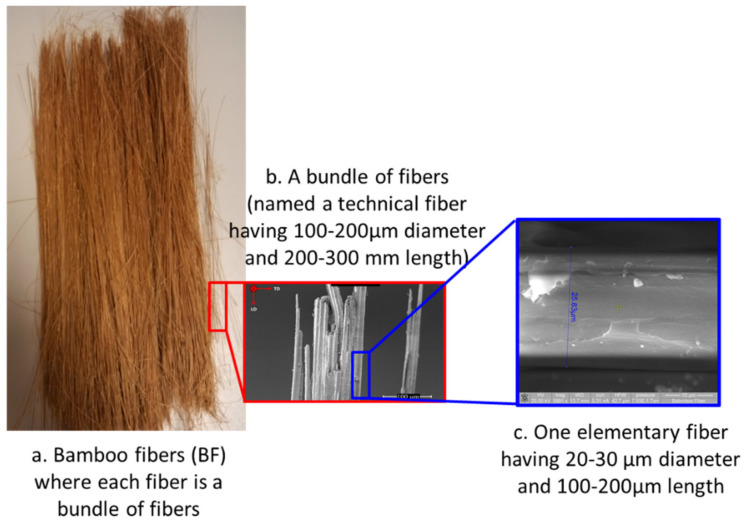
Illustration of bamboo fibers extracted using U.S. Patent No.10184215, optical microscope image of a bundle of fibers and scanning electronic microscopy of one elementary fiber.

In an elementary bamboo fiber, cellulose microfibrils are surrounded by lignin-carbohydrate complex matrices that mainly contain lignin and hemicellulose with volumetric percentages of 10.50% and 12.49%, respectively. A bundle of fibers is represented by a certain number of elementary fibers which contains a higher percentage of the lignin-carbohydrate complex matrix due to the limitation of the extraction process to dissolve the lignin between the elementary fibers. An illustration of the BFs, a bundle of fibers and an elementary fiber is presented in Figure 1.

### 2.2. Chemically-Induced Surface Modification of the Fibers

A common characteristic of natural fibers, including BFs, is a low bond with synthetic polymers caused by a passive interaction between hydrophilic BFs and a hydrophobic polymeric matrix. This results in a weak adhesion and consequently leads to less performance of the composite mechanical properties, such as composite strength, stiffness, toughness [1,8]. Hence, chemical treatments are necessary to improve the compatibility of the matrix and fibers either by modifying the polymeric matrix, such as by the addition of maleic anhydride (MA) as a compatibilizer, or applying a chemical treatment to the BFs, such as coupling agents [7,12,13]. Coupling agents react with hydroxyl groups (or others functional groups) of natural fibers and with functional groups of the matrix; thus, forming bridges of chemical bonds between the fibers and the matrix.

Among the coupling agents, silanes have a hydrophilic structure with different groups attached to the silicon atom where one end interacts with the matrix and the other one interacts with the hydrophilic fiber (e.g., BFs) [13,14]. The MA polar groups also form covalent and hydrogen bonds with the surface of the BFs, which improve the BF-matrix adhesion [7,12].

Based on these findings, two approaches were proposed to chemically functionalize the fibers interface, namely: (1) immersion of the fibers in a coupling agent and then combining them with the polymer matrix, and (2) addition of a compatibilizer to the polymer matrix. To increase the adhesion of the BFs to the polymeric matrix, as described above, the BFs were chemically treated by immersion in an 5% NaOH solution and then washed with distilled water until a neutral pH was realized. The resulting BFs, denoted as BFs^AL^ (AL-alkaline treatment), were then further chemically treated by immersion into a tetramethoxy orthosilicate (TMOS, Sigma-Aldrich, St. Louis, MO, USA) coupling agent, denoted as BFs^TMOS^. For the TMOS silane coupling agent, TMOS was hydrolyzed in a methanol solution (Aqua Phoenix Scientific) with 40% water content at pH 6–7. Once hydrolysis was complete, the BFs^AL^ were immersed in the solution to allow the silane coupling to react. BFs^TMOS^ was incorporated with a polyethylene-graft-MA (Sigma-Aldrich) compatibilizer resulting in BFs^MA^. The resulting BFs, denoted BFs^TMOS^ and BFs^MA^ were finally air-dried after the coupling reaction was complete.

### 2.3. Bamboo Fiber Reinforced Composite Sheet Fabrication

A silane coupling agent and MA compatibilizer was also utilized to develop composite sheets by enhancing the LDPE-BFs interfacial strength. Thus, three categories of the prepared fibers were used in manufacturing composite sheets, as follows:

1. LDPE-BFs^AL^: LDPE reinforced with extracted BFs subjected to alkaline treatment;
2. LDPE-BFs^TMOS^: LDPE reinforced with alkaline-treated BFs immersed in a TMOS coupling agent;
3. LDPE-BFs^MA^: LDPE with added MA compatibilizer reinforced with BFs^TMOS^.

**Figure 2:**
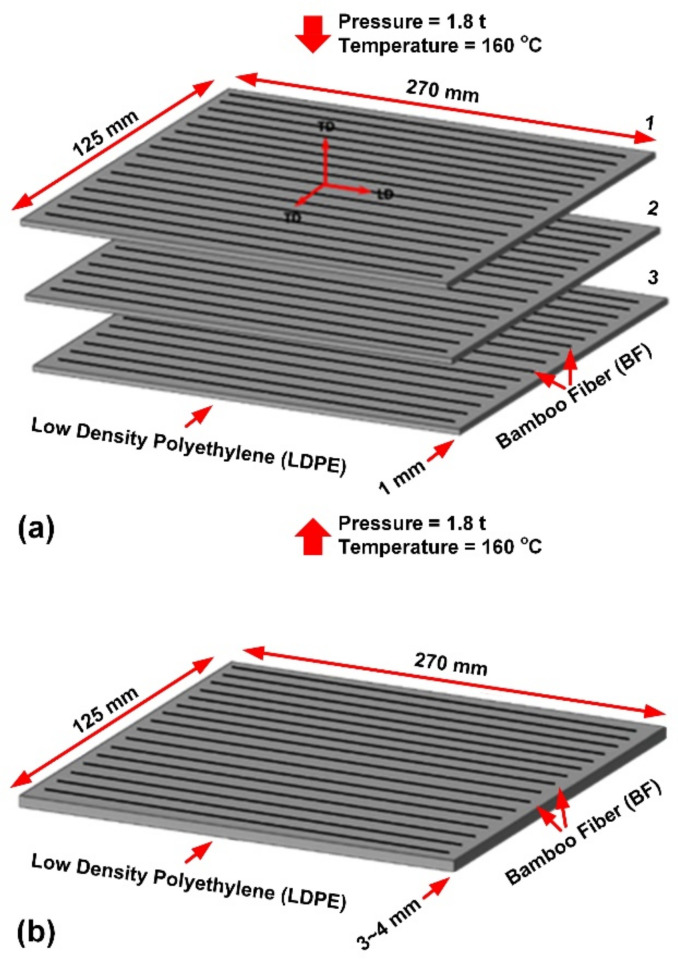
Dimensions of the prepregs and an example of the composite sheets used for further characterization, (**a**) prepregs in unidirectional plies and (**b**) an example of a composite sheet [0]_3_.

To prepare the necessary single-layer fibers (prepregs) to develop multi-ply LDPE-BFs composite sheets (40% BFs and 60% LDPE, vol. %), BFs and LDPE pellets (melt index: 6.5 g/10 min; Marco Polo International) were compressed in a mold using a hand lay-up process, then heated in an air oven at 160 °C for 1 h. The mixture of fibers (BFs) and a matrix (LDPE) was then hot-pressed at the same temperature and a pressure of up to 1.8 t and subsequently air-cooled to ensure completion of the polymer curing process. Three developed prepregs of 270 mm × 125 mm × 1.0 ± 0.3 mm were finally added together in a unidirectional configuration [3] (see Figure 2) and subjected to a similar pre-heating and compression molding process to develop the final 40 vol. % BF-reinforced LDPE composite sheets.

To ensure a complete immersion of the fibers in the matrix, the fiber-matrix interface was analyzed through microscopy of the composite sheet for LDPE-BFs^AL^. Samples of 10 mm × 10 mm × 3 mm were cut from the composite sheet and studied using scanning electronic microscopy (SEM).

### 2.4. Methods for Experimental Testing

#### 2.4.1. Three-Point Bending Testing

To assess the stiffness, strength and toughness of the [0]_3_ composite, a three-point bending test was performed for determination of the flexural behavior and further investigation of the fracture mechanism. The tests were performed according to ASTM D 790-7 standard, using a material test system (MTS 550 R) at a length-to-depth ratio of 32 and strain rate of 10 mm/min. LDPE-BFs^AL^, LDPE, LDPE-BFs^TMOS^ and LDPE-BFs^MA^ combination of fiber and matrix were used for sample preparation. To ensure the validity of the results, four replicates were used for each type of fiber-matrix combination.

The flexural strength (σ_f_), flexural modulus (E_f_), and strain-to-failure (ε*_f_*) of each strip were evaluated from the measured load-displacement data as follows [14]:

(1) $$\sigma_{f}=\frac{3P_{Max}L}{2bh^{2}}[1+6{\left(\frac{D}{L}\right)}^{2}-4(\frac{h}{L})(\frac{D}{L})]$$

(2) $$E_{f}=\frac{mL^{3}}{4bh^{3}}$$

(3) $$\varepsilon_{f}=\frac{6Dh}{L^{2}}$$

where *L*, *b*, and *h* are the length, width, and depth of the specimen, respectively. *P*_max_ is the maximum recorded load, *m* is the slope of the tangent to the initial straight portion of the load–deflection curve, and *D* is the maximum deflection before the strip failure.

To further explore the impact of the chemical treatment on the bonding strength, stress intensity factor *K*_I_ determined from the mode I fracture of different composite sheets was calculated by tensile testing with a material test system (MTS 550) on pre-notched specimens at a strain rate of 3 mm/min. The *K*_I_ of each composite was calculated using Equation (4) [15]:

(4) $$K_{I}=\sigma \sqrt{\pi a}[1.12-0.23(\frac{a}{b})+10.6{\left(\frac{a}{b}\right)}^{2}-21.7{\left(\frac{a}{b}\right)}^{3}+30.4{\left(\frac{a}{b}\right)}^{4}]$$

where σ is the composite failure stress, *a* is the length of the induced edge crack, and *b* is the width of the specimen.

#### 2.4.2. Dynamic Mechanical Analysis

A dynamic mechanical analysis (DMA) using a cyclic tensile test scheme was performed on the three types of bamboo composite samples for determination of the loss modulus (E”, GPa), the storage modulus (E’, GPa), tan delta which represents the ratio between loss and storage moduli used in assessing the damping of the composite, and glass transition temperature (T_g_). The samples were analyzed using the dynamic temperature ramp method at a heating rate of 5 °C/min using the TA Instrument RSA3 DMA (TA, New Castle, DE, USA).

## 3. Results and Interpretations

### 3.1. Microstructure of the LDPE-BFs^AL^ Composite Sheet

**Figure 3:**
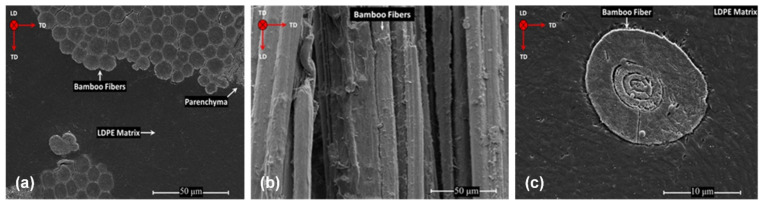
(**a**) Representative SEM image displaying the morphology of the LDPE-BFs^AL^ composite sheet, (**b**) longitudinal view of the bamboo bundles and (**c**) high-magnification image showing the interfacial area of an individual BFs^AL^ with the LDPE matrix in the LDPE-BFs^AL^ composite sheet.

Figure 3 shows the microstructure of the LDPE-BFs^AL^ composite sheet, indicating a complete wetting of the fibers with the LDPE matrix. As shown in Figure 3b, the longitudinal view of the fibers shows a roughness surface of the fibers, which is induced by the alkaline treatment. Further, only one elementary fiber was isolated from the composite sample and analyzed with a higher magnification (Figure 3c) showing in detail the morphology of the bamboo elementary fiber and a complete wetting of the fiber in the matrix.

**Figure 4:**
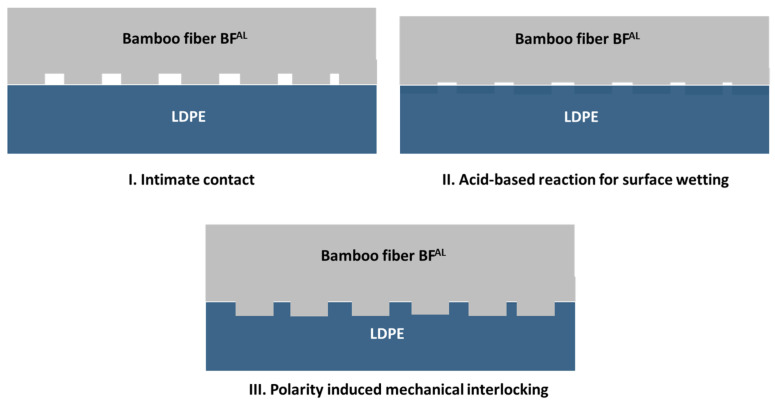
Fiber-matrix interfacial bonding mechanism: (**I**) intimate contact, (**II**) acid-based reaction and surface wetting, (**III**) polarity-induced mechanical interlocking.

Based on the microscopically observed interface between the fibers and the matrix, it is assumed that the bonding mechanism between the BFs^AL^ and LDPE is composed of three phases, presented in Figure 4.

Phase I, intimate contact; when the surface irregularities of the fibers such as roughness are in contact with the polymer chains. Phase II, acid-based reaction and surface wetting; when the acid-base reaction that occurred between the Lewis base (NaOH) and acid (cellulose in the BFs) during the alkali treatment forming a sodium alkoxide compound, which, along with the O-Na^+^ groups, can play a significant role in expanding the dimension of the cellulose molecules. Phase III, polarity-induced mechanical interlocking; when mechanical interlocking is formed through a change in the polarity of the surface of the BFs and by removing amorphous cellulose from the cellulose fibers, which consequently increases the roughness of the BFs [12]. BFs with a rougher surface can more easily maintain mechanical interlocking with LDPE since during prepreg development, the polymer melt can simply penetrate the predeveloped pores and crevices.

### 3.2. Influence of Chemically Functionalization on Mechanical Properties

#### 3.2.1. Evaluation of the Bonding Strength

**Figure 5:**
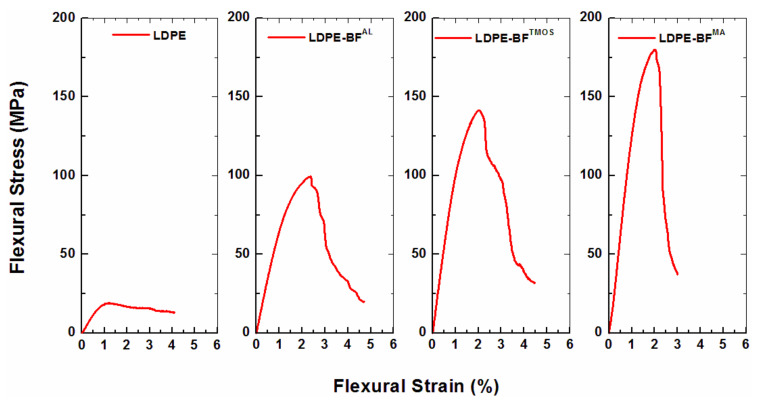
The flexural stress of neat LDPE and different LDPE-BFs composite sheets.

The static tests applied to LDPE-BFs^AL^, LDPE-BFs^TMOS^ and LDPE-BFs^MA^ composite sheets demonstrates that the incorporation of the long BFs^AL^ into the neat LDPE matrix substantially improved the flexural stress (Figure 5) and flexural stiffness (*E*_f_), and flexural strength (σ_f_) and toughness compared to neat LDPE. As shown in Figure 5, further improvement of the flexural stress was also observed when the BFs^TMOS^ were incorporated into the LDPE matrix. Among the developed composite sheets, the largest improvement in flexural stress was achieved when MA was added to the mixture of BFs^AL^ and LDPE.

**Figure 6:**
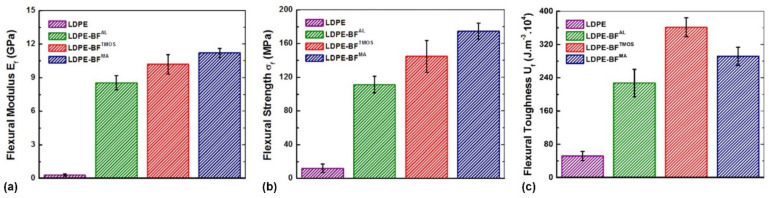
The influence of the alkaline treatment and TMOS and MA coupling agents on the (**a**) flexural modulus, (**b**) flexural strength and (**c**) flexural toughness compared to the neat LDPE.

Along with the enhancement in flexural stiffness (*E*_f_) and flexural strength (σ_f_) of the LDPE-BFs, a substantial improvement in the flexural toughness (*U*) of the LDPE-BFs composite sheets compared to that of neat LDPE was also observed, as shown in Figure 6. The incorporation of the BFs^AL^ into the neat LDPE matrix significantly increased the flexural toughness of LDPE, and further improvement was achieved when the BFs were treated with TMOS and MA. This enhanced *U* of the LDPE-BFs is due to the enhanced strength of the composites and not the ductility. The LDPE-BFs composite sheets demonstrated similar or even slightly lower (in the case of LDPE-BFs^MA^) strain-to-failure (*ε*_f_) compared to those of neat LDPE (see Figure 6).

#### 3.2.2. Evaluation of the Toughness

Although the increase in the flexural response of the LDPE-BFs^AL^ composite sheet compared to neat LDPE is partially justified by the incorporation of tougher BFs (Young’s modulus 30.1 ± 3.0 GPa) [16] into the soft LDPE matrix (Young’s modulus 0.83 ± 0.54 GPa), the indirect contribution of toughening mechanisms arising from the presence of long BFs should not be neglected. Long BFs increased the flexural response of the LDPE-BFs composite sheets via resistance to both crack initiation (i.e., constant crack length) and propagation (i.e., increasing crack length); the crack bridging induced by the bridging of the BFs and the crack deflection mechanisms were attributed to the former and latter mechanisms, respectively.

**Figure 7:**
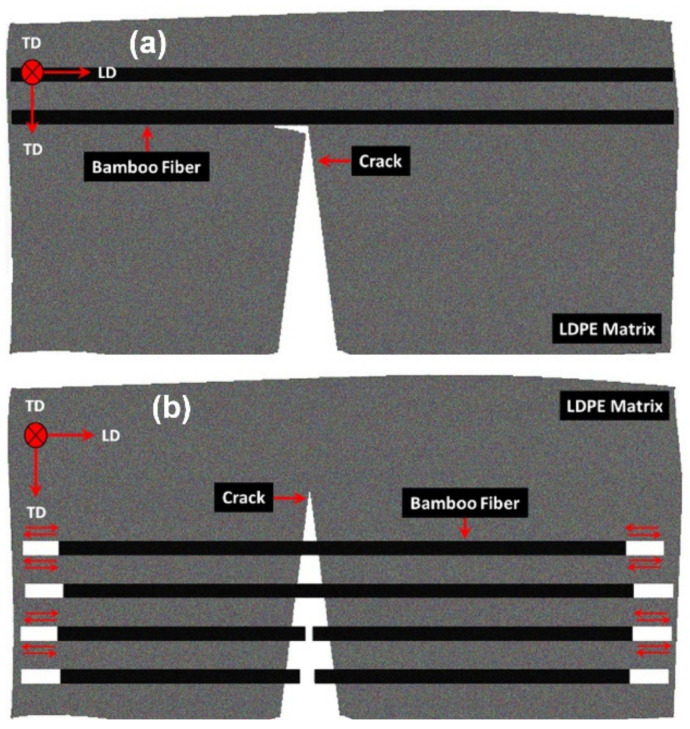
Schematic representation of crack (**a**) bridging and (**b**) deflection mechanisms triggered by the presence of long BFs in the LDPE-BFs composite sheets.

In terms of the crack bridging mechanism, as demonstrated in Figure 6a, the separation of a matrix crack bridged by uniaxial aligned BFs requires the matrix to slip over the BFs [17]. However, slipping is restricted by frictional forces that lead to a reduction in the crack surface displacement, which is equivalent to applying closure stress to the crack surface (see Figure 7a). Hence, owing to crack bridging, higher loads must be applied to initiate the crack. This reduction in the stress intensity factor at the crack tip can be expressed as [18]:

(5) $$K_{eff}^{B}=K_{a}-K_{s}$$

where $K_{eff}^{B}$ is the effective stress intensity factor at the crack tip [MPa·m^1/2^], owing to the crack bridging mechanism, *K*_a_ is the applied stress intensity factor [MPa·m^1/2^], and *K*_s_ is the shielding stress intensity factor [MPa·m^1/2^], which depends upon the interfacial strength of the LDPE-BFs.

In terms of the crack deflection mechanism, it was also demonstrated that the BFs can tailor the growth of cracks during their propagation (see Figure 7b). It was supposed that the LDPE-BFs composites mostly experienced mode I crack opening during loading. Hence, any deviation of the crack away from the mode I growth plane (straight crack growth) can lead to a mixed loading mode at the crack tip, which would reduce the overall driving force for further crack propagation. In terms of the geometry-dependent model developed by Suresh et al. [19,20], presuming that the crack kinked at an average incline θ with respect to the mode I plane, the local effective stress intensity factor at the crack tip can then be estimated by [20]:

(6) $$K_{eff}^{D}=\cos^{2}(\frac{\theta }{2})K_{a}$$

where $K_{eff}^{D}$ is the effective stress intensity factor at the crack tip owing to the crack deflection mechanism [MPa·m^1/2^], *K*_a_ is the applied stress intensity factor [MPa·m^1/2^], and *θ* is the deflection degree. It is worth mentioning that the contribution of the crack deflection mechanism is more prominent when crack growth occurs along the thickness of the strips. Additionally, the BFs within the LDPE can trigger crack deflection toughening once the interface of the LDPE-BFs is strong enough to avoid any delamination before the start of crack nucleation and growth.

#### 3.2.3. Toughening Mechanism

**Figure 8:**
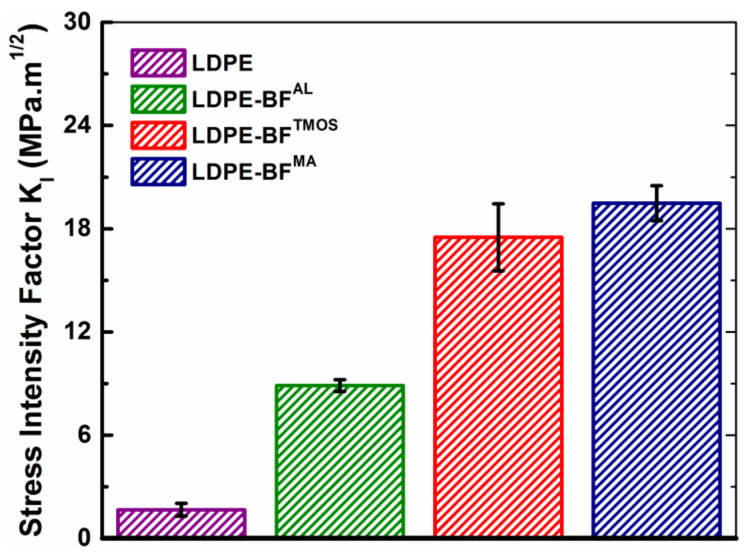
Variation in mode I stress intensity factor (*K*_I_) of LDPE and LDPE-BFs composite sheets vis-à-vis BFs chemical treatment.

With regard to the BF-induced toughening mechanisms, the extent of the contribution of the crack bridging mechanism would vary depending on the chemical treatment of the BFs, whereas any significant change in the contribution of the crack deflection mechanism with the BFs chemical treatment would be unlikely. As shown in Figure 8, the LDPE-BFs composite sheets reinforced with different chemically treated BFs exhibited different Mode I stress intensity factors (*K*_I_), which is partially attributable to the sensitivity of the crack bridging mechanism associated with the interfacial strength. The incorporation of the BFs^AL^ into the LDPE matrix significantly increased the *K*_I_ of LDPE by triggering the aforementioned crack bridging and deflection mechanisms. The significant increase in *K*_I_ of the LDPE-BFs^TMOS^/BFs^MA^ composite sheets (see Figure 8) induced by the addition of TMOS and MA, respectively, was also attributable to the increased shielding stress intensity factor (*K*_s_), which was caused by the established covalent bonds between the BFs^TMOS^/BFs^MA^ and LDPE. The estimated increase in *K*_s_ attributed to this chemical bonding would decrease the effective stress intensity factor$\;K_{\mathrm{eff}}^{B}$, which would consequently increase the *K*_a_ needed to maintain the deformation (see Equation (5)).

**Figure 9:**
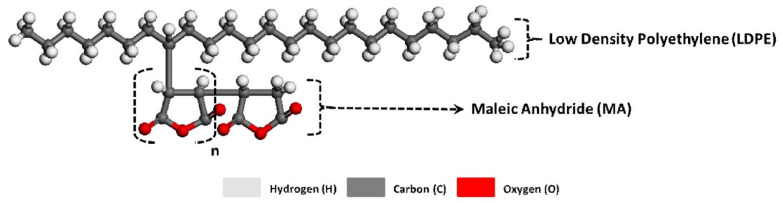
Molecular configuration of chemical reactions between the MA and LDPE in the case of LDPE-BFs^MA^ composite sheet. Here, n represents the oligomerization degree of MA.

In addition to increasing the interfacial strength of the LDPE-BFs by acting as a bonding agent, MA also contributed to strengthening the LDPE-BFs^MA^ composite sheets by changing the properties of the LDPE matrix. As depicted in Figure 9 once MA was added to the LDPE, the MA free radical grafted to the LDPE; thus, changing its molecular weight. Once the oligomerization reaction of MA took place, a larger chain of MA was grafted onto LDPE, which further increased the molecular weight and consequently the strength of the LDPE matrix [21]. The stress intensity factor was obtained with an error of ±15% for LDPE-BFs^TMOS^ and ±7% for LDPE-BFs^MA^. The source of error is the uncertainty in the number of the elementary fibers included in a bundle and consequently, the intimate contact between the LDPE and the fibers has variations. This level of error is acceptable for the natural fiber composites knowing that the plant growth is depended on environmentally uncontrolled factors.

#### 3.2.4. Evaluation of the Failure Mode

**Figure 10:**
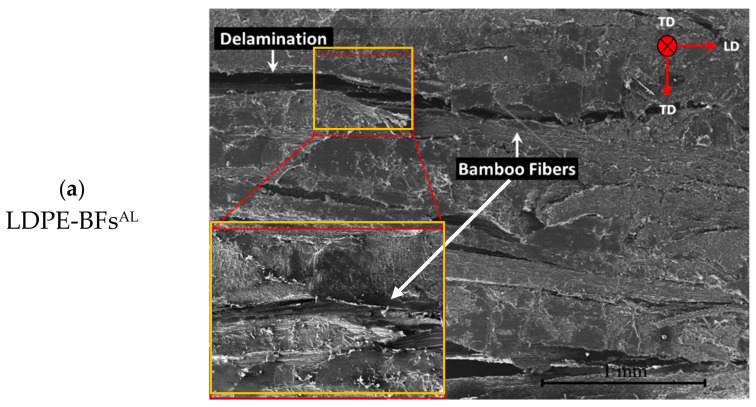

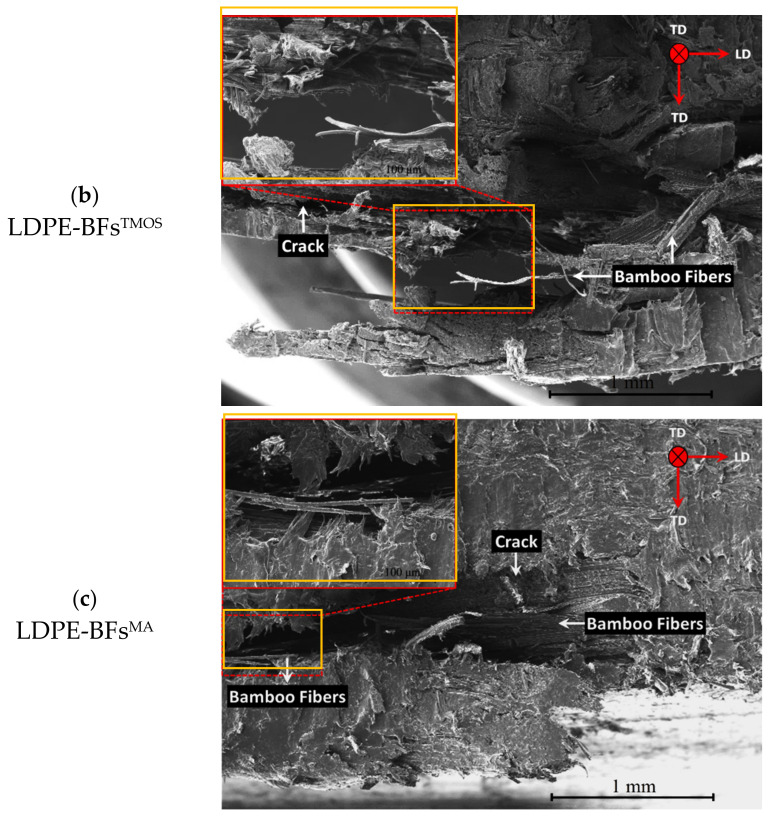
SEM images showing the fracture modes subjected to flexural loading: (**a**) fracture of LDPE-BFs^AL^ shows a delamination of the matrix longitudinal to the fiber, (**b**) fracture of LDPE-BFs^TMOS^ shows pullout of fibers from the matrix and a ductile fracture of the matrix, and (**c**) fracture of LDPE-BFs^MA^ shows pullout of fibers from the matrix, crack propagation in the matrix and ductile fracture of the matrix.

Along with the quantitative evaluations, the enhanced interfacial strength of the BFs^TMOS^/BFs^MA^ with the LDPE matrix was also qualitatively evaluated based on the exhibited fracture mechanisms for each composite subjected to flexural loading. It is expected that, compared to the BFs^AL^, the improved adhesion between the BFs^TMOS^/BFs^MA^ and LDPE would enable a greater stress transfer between the fibers and matrix, which would accordingly reduce the chance of BFs de-bonding. As such, the BFs would share a larger load with the matrix and thereby increase the *K*_I_ of the composite sheet, as discussed quantitatively earlier, by reducing the extent of the BFs pullout, which is a major source of energy dissipation. As shown in Figure 10a, the LDPE-BFs^AL^ composite sheet subjected to flexural loading failed via delamination between the BFs^AL^ and LDPE. The exhibited fracture mode clearly demonstrated that despite the development of mechanical interlocking, the interface of the LDPE-BFs^AL^ was not strong enough to avoid delamination before the nucleation and growth of the transversal crack. On the contrary, the LDPE-BFs^TMOS^/BFs^MA^ composite sheets demonstrated different failure modes when subjected to flexural loading. As shown in Figure 10b, unlike the dominant delamination in the case of LDPE-BFs^AL^, the crack propagated across the sheet thickness following the crack bridging and deflection mechanisms in the case of LDPE-BFs^TMOS^. This failure mode clearly confirmed that, due to the presence of the TMOS coupling agent, the LDPE-BFs^TMOS^ interface was sufficiently strengthened to not fully accommodate delamination and crack propagation. The displayed failure mode of the LDPE-BFs^MA^ composite sheet (see Figure 10c) unveiled its brittle nature, as demonstrated by the rapid propagation of the crack through the specimen without necessarily following any specific route once it had nucleated.

To explore the dispersion of BFs in conjunction with their interfacial areas with the LDPE matrix, microstructural characterization was conducted on polished samples taken from the cross-sections of the composite sheets. The fracture surfaces of different composite sheets subjected to flexural loading were also investigated. For this purpose, scanning electron microscopy (SEM, Philips XL30 FEG, Philips, Eindhoven, The Netherlands) was conducted.

The strong adhesion between fibers and matrix in the LDPE-BFs^TMOS^ composite sheet, as proved by the static mechanical tests (Figure 5 and Figure 6), can also be attributed to the chemical bonds formed between the BFs^TMOS^ and LDPE matrix. Once the BFs^AL^ were placed in the TMOS solution, the reaction between the Lewis base (TMOS) and acid (cellulose in the BFs) led to the formation of silanol by hydrolyzing the alkoxy (OH) and methoxy (OCH_3_) groups from cellulose and TMOS, respectively [9]. Once silanol formed, TMOS was linked with BFs through the creation of a covalent bond between the Si and O atoms from TMOS and cellulose, respectively. Silanol then induced bonding between the BFs and LDPE during prepreg development by establishing covalent bonds between the Si and C atoms of the LDPE.

Similar to the TMOS coupling agent, the MA compatibilizer increased the LDPE-BFs interfacial strength by acting as a bonding agent and establishing covalent bonds between the BFs and LDPE. During prepreg development, because the free radical of LDPE can freely associate, the C=C bonds in the MA structure break, resulting in the formation of C-C bonds between not only two MA C atoms but also C atoms from MA and LDPE. The O atom from MA also forms a covalent bond with the C atom from the BFs cellulose, which eventually leads to adhesion between the BFs and LDPE. This bonding between MA and cellulose is the result of a nucleophilic reaction between their C=O and OH groups, respectively, which was observed as the loop opening of the MA.

Regarding the demonstrated mechanical and chemical bonding of the BFs^AL^ and BFs^TMOS^/BFs^MA^ with LDPE, it is reasonable to speculate that the LDPE-BFs interface is stronger in the LDPE-BFs^TMOS^/BF^MA^ composite sheets than in the LDPE-BFs^AL^ sheets. The inconsistent interfacial strength of LDPE with the BFs^TMOS^ and BFs^MA^, as demonstrated by the different flexural responses of LDPE-BFs^TMOS^/BFs^MA^ (see Figure 6), can also be attributable to dissimilarities in the energy of the bonds established by TMOS and MA. The Si atom in the TMOS coupling agent established covalent bonds with the C and O atoms of LDPE and cellulose, respectively. The bonding energies of Si-C (TMOS-LDPE) and Si-O (TMOS-BF) are 318 and 428 KJ/mol [22], respectively. On the contrary, the C and O atoms in the MA compatibilizer created separate covalent bonds with C atoms from LDPE and BF; the bonding energies of C-C (MA-LDPE) and O-C (MA-BF) are 346 and 358 KJ/mol [22], respectively. In terms of bonding energies, it is expected that during deformation of the composite sheets, the Si-C (TMOS-LDPE) and C-C (MA-LDPE) bonds were more susceptible to failure, and the difference in their bonding energies can justify the difference in the interfacial strengths of the LDPE-BFs^TMOS^/BFs^MA^ composite sheets.

### 3.3. Influence on the Viscous Behavior

**Figure 11:**
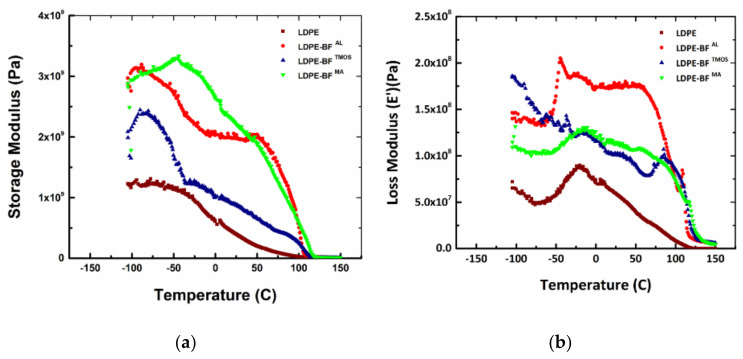
(**a**) Storage modulus and (**b**) loss modulus of LDPE, LDPE-BFs^AL^, LDPE-BFs^TMOS^ and LDPE-BFs^MA^.

The incorporation of bamboo fibers prevents the mobility of polymer chains, decreases the loss modulus, decreases the damping of the composite and shifts T_g_ toward a higher temperature through restricting the molecular motion in the matrix. The error in the temperature is ± 2 °C. A polymer composite has a viscoelastic behavior for which the elastic modulus has a complex form. This complex form has a real part expressed through the storage modulus responsible for the elastic part of the behavior, and an imaginary part expressed through the loss modulus which indicates the energy dissipated as heat. Through a DMA tests, storage and loss moduli are determined as the function of temperature. By representing the storage modulus versus temperature and loss modulus versus temperature, the T_g_ and T_m_ can be observed. The first inflection point of the curve indicates T_g_ and the second one indicates T_m_. The fibers perform an important role in alteration the storage and loss moduli. As presented in Figure 11a, the LDPE curve shows the lowest storage modulus compared to the LDPE-BFs^AL^, LDPE-BFs^TMOS^ and LDPE-BFs^MA^. A significant increase (3 times) in the storage modulus is observed in the case of LDPE-BFs^MA^ associated with an increased T_g_. This increase is in agreement with the results from the static testing where this combination resulted in higher stiffness. On the other hand, the loss modulus (Figure 11b) indicates that the most significant influence of the fibers is provided by the LDPE-BFs^AL^ which explains the failure mode seen in Figure 11a.

**Figure 12:**
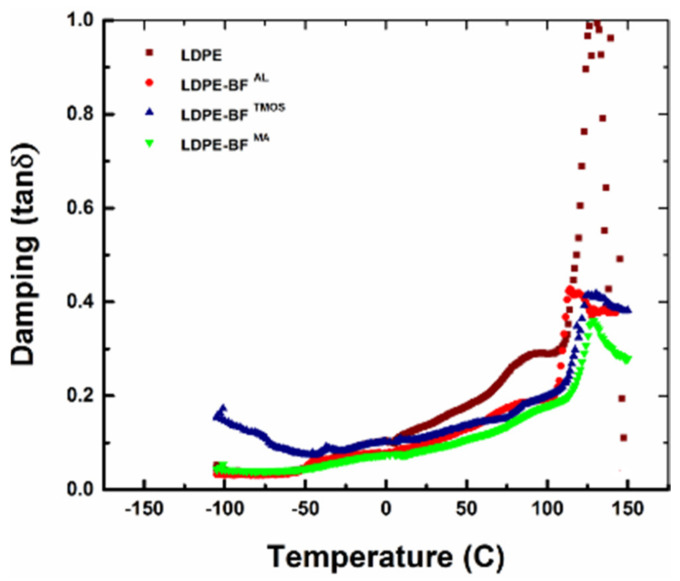
Damping evolution of LDPE, LDPE-BFs^AL^, LDPE-BFs^TMOS^ and LDPE-BFs^MA^.

The two plots of the storage and loss modulus depending on the temperature are used to calculate the damping of the polymer composite, which is expressed as the ratio between the loss modulus and storage modulus, also called tanδ. The dependence of  tanδ to the temperature indicates the evolution of the material damping. When  tanδ is closer to 1, the material has the energy dissipated high and the viscous component is dominant. As seen in Figure 12,  tanδ for LDPE-BFs^AL^, LDPE-BFs^TMOS^ and LDPE-BFs^MA^ is much lower, indicating that the elastic behavior is dominant. It can be concluded that the addition of the fibers increases the capacity of the material to sustain loadings and increases the damping of LDPE-BFs^TMOS^ for lower temperature compared to the LDPE. The key values of the determined damping for the LDPE, LDPE-BFs^AL^, LDPE-BFs^TMOS^ and LDPE-BFs^MA^ are summarized in Table 1.

**Figure 13:**
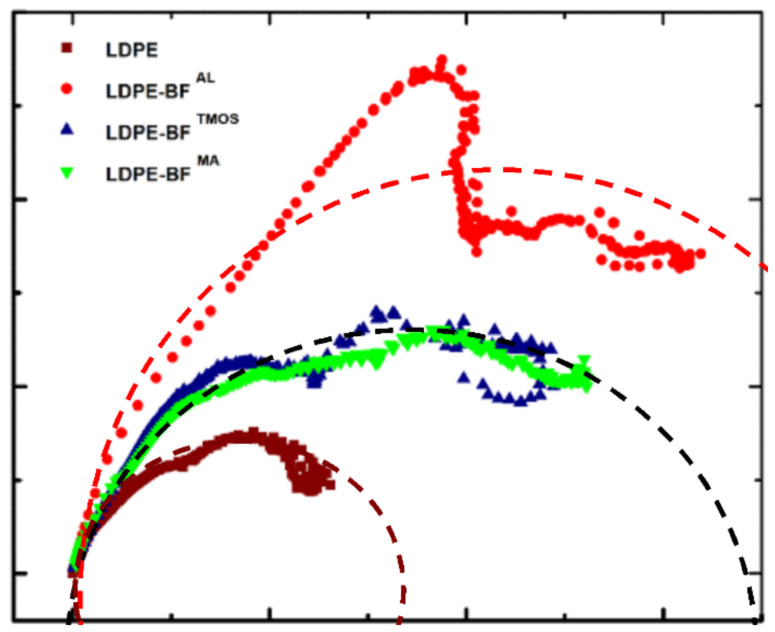
Cole-Cole plots for LDPE with different coupling agents and bamboo fibers; indicator for durability for composite; dot semicircles represent the analytical models of the Cole-Cole plots using h and k parameters [23] for each combination of LDPD and BFs. Deviation from the semicircle qualitatively indicates the anisotropy produced by the fibers and the coupling agent.

The anisotropic behavior of a material is higher when the deviation forms a semicircle plot of the loss vs. storage modulus when storage is a higher Cole-Cole plot, as shown in Figure 13. Thus, the effect of the fibers on the rheology behavior of the LDPE is studied, concluding that the isotropic behavior of the LDPE is modified by the addition of the fibers generating an anisotropic behavior. It is noticed that MA coupling agent generates the higher anisotropy compared to the TMOS and MA which generates a medium anisotropy of the LDPE-BFs^MA^ and LDPE-BFs^TMOS^ composites. By analyzing this plot, it is observed that LDPE-BFs^MA^ and LDPE-BFs^TMOS^ is more homogeneous than LDPE-BFs^AL^ and consequently lead to a higher durability of the composite products by avoiding delaminations (as shown in Figure 11a).

## 4. Conclusions

Significant improvement in the flexural response of LDPE was achieved by combining LDPE with long BFs through a compression molding process. In addition to their superior mechanical behavior, long BFs can also enhance the flexural response of neat LDPE by resisting crack initiation and propagation, as evidenced by the fact that the former and latter mechanisms were reliant on and independent of, respectively, the interface of the LDPE-BFs. Further improvement in the flexural response of the composite sheets was realized by improving the LDPE-BFs interface through the addition of TMOS as a coupling agent and MA as a compatibilizer. The enhanced interfacial strength increased and decreased the shielding stress intensity factor and local stress intensity factor, respectively, at the crack tip.

The additives generate a series of covalent bonds between the bamboo fibers and low-density polyethylene [24], which decreases the local stress intensity factor by increasing the shielding stress intensity factor related to the crack bridging mechanism.

A significant increase in the flexural response of the bamboo fiber composite is obtained compared to the neat polymer due to the partial triggering of crack bridging and the deflection mechanisms and an improved damping of the composite. Furthermore, the addition to a coupling agent, a maleic anhydride compatibilizer further enhanced the flexural response of the composite sheets by increasing the interfacial strength of the bamboo fiber-reinforced composite.

## 5. Patents

Patent used in this paper U.S. Patent No. 10,184,215 issued on 12/2018, Natural fiber reinforced composite panels, Inventors: M. Banu, J. S. Hu, T. Kim, S. Young.

## Figures and Tables

**Table 1:** Damping coefficient and glass transition temperature of neat LDPE and the LDPE-BFs composites sheets.

Materials	Damping Coefficient	Glass Transition Temperature (T_g_) (°C)
LDPE	0.068	~−20
LDPE-BFs^AL^	0.052	~60
LDPE-BFs^TMOS^	0.048	~69
LDPE-BFs^MA^	0.042	~75

## Data Availability

The data presented in this study are available on request from the corresponding author.
